# Activated Chicken Gamma Delta T Cells Are Involved in Protective Immunity against Marek’s Disease

**DOI:** 10.3390/v15020285

**Published:** 2023-01-19

**Authors:** Ayumi Matsuyama-Kato, Bahram Shojadoost, Nitish Boodhoo, Sugandha Raj, Mohammadali Alizadeh, Fatemeh Fazel, Charlotte Fletcher, Jiayu Zheng, Bhavya Gupta, Mohamed Faizal Abdul-Careem, Brandon L. Plattner, Shahriar Behboudi, Shayan Sharif

**Affiliations:** 1 Department of Pathobiology, Ontario Veterinary College, University of Guelph, Guelph, ON N1G 2W1, Canada; 2 Ceva Animal Health Inc., Research Park Centre, Guelph, ON N1G 4T2, Canada; 3 Faculty of Veterinary Medicine, University of Calgary, Calgary, AB T2N 1N4, Canada; 4 Department of Diagnostic Medicine/Pathobiology, College of Veterinary Medicine, Kansas State University, Manhattan, KS 66506, USA; 5 The Pirbright Institute, Pirbright, Woking, Surrey GU24 0NE, UK

**Keywords:** chicken, γδ T cell, Marek’s disease virus, adoptive transfer, cytokine, cytotoxicity

## Abstract

Gamma delta (γδ) T cells play a significant role in the prevention of viral infection and tumor surveillance in mammals. Although the involvement of γδ T cells in Marek’s disease virus (MDV) infection has been suggested, their detailed contribution to immunity against MDV or the progression of Marek’s disease (MD) remains unknown. In the current study, T cell receptor (TCR)γδ-activated peripheral blood mononuclear cells (PBMCs) were infused into recipient chickens and their effects were examined in the context of tumor formation by MDV and immunity against MDV. We demonstrated that the adoptive transfer of TCRγδ-activated PBMCs reduced virus replication in the lungs and tumor incidence in MDV-challenged chickens. Infusion of TCRγδ-activated PBMCs induced IFN-γ-producing γδ T cells at 10 days post-infection (dpi), and degranulation activity in circulating γδ T cell and CD8α^+^ γδ T cells at 10 and 21 dpi in MDV-challenged chickens. Additionally, the upregulation of IFN-γ and granzyme A gene expression at 10 dpi was significant in the spleen of the TCRγδ-activated PBMCs-infused and MDV-challenged group compared to the control group. Taken together, our results revealed that TCRγδ stimulation promotes the effector function of chicken γδ T cells, and these effector γδ T cells may be involved in protection against MD.

## 1. Introduction

Gamma delta (γδ) T cells are a T cell subset which expresses the heterodimer antigen receptor γ and δ chains. These cells have a unique feature as their functions are associated with both innate and adaptive immunity [1]. Gamma delta T cells are involved in immunity against pathogens, tumor control, immune surveillance, and homeostasis [2,3,4]. Although the role of γδ T cells in human immune responses is well defined, the function of these cells in the control of pathogens in chickens is yet unclear. Recently, we reported the phenotypic characterization of defined chicken γδ T cell subsets, interferon (IFN)-γ^+^ γδ T cells, transforming growth factor (TGF)-β^+^ γδ T cells, and cytotoxic γδ T cells in chickens infected with Marek’s disease virus (MDV) [5]. However, the role of γδ T cells in the control of MDV was yet unknown.

Marek’s disease (MD) is a malignant lymphomatous disease in chickens that is caused by pathogenic strains of MDV. MDV is a cell-associated virus, but cell-free viruses are produced and shed from the feather follicle epithelium of MDV-infected birds. Cell-free viruses are present in feather dander, which acts as the main vehicle for the transmission of MDV from infected to other susceptible birds [6]. Inhaled cell-free virus particles are thought to infect lung epithelial cells and antigen-presenting cells, and subsequently, lung-infiltrating B and T lymphocytes become targets of MDV [7,8]. MDV leads to lysis of the infected lymphocytes at the early cytolytic infection phase (3–7 days post-infection (dpi)) and latent infection at the latency phase (7–14 dpi) [9]. MDV is reactivated preferentially in CD4^+^ T cells at the second cytolytic infection phase (14–28 dpi), and MDV-infected CD4^+^ T cells are transformed to lymphoma cells, which proliferate and form solid tumors in various organs. To protect chickens from MD, the cell-mediated immune response is believed to be crucial. To date, the involvement of innate immune system cells including macrophages, natural killer (NK) cells and γδ T cells, and adaptive immune cells including CD4^+^ αβ T cells and CD8^+^ αβ T cells in the immune reaction against MDV infection has been suggested [5,10,11,12].

In our previous report, we demonstrated that MDV vaccination induced IFN-γ^+^ γδ T cells and membrane-bound TGF-β (mTGFβ)^+^ γδ T cells at the early phase of infection [5]. Furthermore, γδ T cells from MDV-challenged chickens at the later phase of infection showed cytotoxic activity. IFN-γ expression in γδ T cells at the early phase of infection was also confirmed in MDV-challenged chickens and in MDV-vaccinated chickens [13,14]. These studies suggest an effector function of γδ T cells in immune responses against MDV, but the detailed role of γδ T cells in MDV infection is still not clear.

To examine if activated γδ T cells have a protective role against MDV infection or can accelerate MD progression, we used an adoptive cell transfer approach which has been successfully applied for the treatment of cancer in human trials [15]. In human studies, it was reported that γδ T cells expand after stimulation with the anti-T cell receptor (TCR)γδ antibody, and ex vivo-expanded γδ T cells can be used for adoptive T cell immunotherapy [16,17]. In the present study, we infused ex vivo-activated peripheral blood mononuclear cells (PBMCs) with an anti-TCRγδ antibody into chickens and analyzed if this infusion provides protection against MD and how immune responses during MDV infection are altered by the infusion of TCRγδ-activated PBMCs. 

## 2. Materials and Methods

### 2.1. Chicken Housing and Ethics

One-day-old specific pathogen-free (SPF) White Leghorn chickens were received from the Animal Disease Research Institute, Canadian Food Inspection Agency (Ottawa, ON, Canada) and were housed in the Campus Animal Facility at the Ontario Veterinary College, University of Guelph. All experiments were approved by the Animal Care Committee of the University of Guelph and were implemented according to the guidelines of the Canadian Council on Animal Care.

### 2.2. Virus Preparation

The very virulent MDV (vvMDV), RB1B strain, was provided by Dr. K.A. Schat (Cornell University, Ithaca, NY, USA) [18]. MDV propagation was conducted in SPF chickens and splenocytes obtained from the infected chickens at 3 weeks post-infection were stored in liquid nitrogen as virus stocks. The virus titers were calculated using primary chicken kidney cells derived from 2- to 3-week-old SPF chickens [19].

### 2.3. Ex Vivo TCRγδ Stimulation

Blood samples were collected from the wing veins of 20-days-old chickens using needles and syringes with 5 mg/mL of heparin (Millipore-Sigma, St. Louis, MO, USA) and diluted with the same volume of phosphate buffered saline (PBS) (Wisent, Inc., St. Bruno, QC, Canada). Diluted blood samples were overlayed onto Histopaque1077 (Sigma Chemical Co., St. Louis, MO, USA) and centrifuged at 560× *g* for 20 min. PBMCs were subsequently aspirated from the interface and washed and centrifuged 3 times at 400× *g* for 5 min in Iscove’s modified Dulbecco’s medium (IMDM) (Gibco, Burlington, ON, Canada) containing 100 U/mL of penicillin and 100 μg/mL of streptomycin (Gibco). Cells were resuspended in complete IMDM cell culture medium: the IMDM medium contained 10% of fetal bovine serum (Millipore-Sigma), 2% of chicken serum (Millipore-Sigma), 100 U/mL of penicillin and 100 μg/mL of streptomycin, 32.7 nM of 2-Aminoethanol (Millipore-Sigma), 1% of Insulin-Transferrin-Selenium (Gibco), 2 μg/mL of linoleic acid (Millipore-Sigma), 2 μg/mL of oleic acid (Millipore-Sigma), 2 μg/mL of palmitic acid (Millipore-Sigma), and 10 ng/mL of recombinant chicken interleukin (IL)-2 protein (Abcam, Cambridge, UK) [20]. Live cell numbers were determined using a haemocytometer and Trypan blue. One million live cells were cultured in immobilized 96-well plates with 10 μg/mL of mouse anti-chicken TCRγδ monoclonal antibody (mAb) (Southern Biotech, Birmingham, AL, USA) (stimulated cells) or antibody-uncoated plates (unstimulated cells) at 41 °C; 5% CO_2_ for 4 h (for degranulation assay); 18 h (for ex vivo-stimulation assay and infusion); or 3, 8, and 18 h (for gene expression analysis). For the ex vivo-stimulation assay, Golgi Plug (Beckton Dickinson Biosciences, San Jose, CA, USA) and 20 μg/mL of DNase I (Millipore-Sigma) were added at 14 h post-stimulation (hps). For the gene expression analysis, the cultured cells were collected and resuspended in 1 mL of Trizol reagent (Invitrogen, Carlsbad, CA, USA).

### 2.4. Experimental Design

One hundred and twenty chickens were randomly divided into 6 groups: PBMCs-uninfused and MDV-unchallenged (control), TCRγδ-unactivated PBMCs-infused and MDV-unchallenged (TCRγδ-/MDV-), TCRγδ-activated PBMCs-infused and MDV-unchallenged (TCRγδ+/MDV-), cell-uninfused and MDV-challenged (MDV+), TCRγδ-unactivated PBMCs-infused and MDV-challenged (TCRγδ-/MDV+), and TCRγδ-activated PBMCs-infused and MDV-challenged groups (TCRγδ+/MDV+). At 21 days of age, 3 × 10^7^ cultured PBMCs in 500 μL of PBS were injected intraabdominally to the original chicken (autologous cell infusion). On the same day of infusion, chickens in the MDV+, the TCRγδ-/MDV+, and the TCRγδ+/MDV+ groups were challenged with 500 plaque-forming units of RB1B intraabdominally. The same volume of PBS was injected as a control for cell infusion and virus challenge to each control group. Five chickens in each group were euthanized by CO_2_ inhalation at 4, 10, and 21 dpi. The spleen and lungs were collected aseptically in 1 × Hank’s balanced salt solution (HBSS) (Gibco) supplemented with 100 U/mL of penicillin and 100 μg/mL of streptomycin and stored on ice until further use. Blood samples were collected using heparinized syringes. Feather tips, spleen, lungs, and PBMCs were collected in RNAlater (Qiagen Inc., Mississauga, ON, Canada) and stored at −20 °C. Tumor incidence and scoring of MDV-challenged chickens were determined at 21 dpi (*n* = 10) [21]. The spleen, lungs, liver, kidneys, heart, genitalia, proventriculus, gizzard, pancreas, intestine, and skin were assessed for gross tumor lesions. Tumor scoring was calculated by the number of tumor-bearing organs. The neoplastic lesion score of chickens which did not have any gross tumor lesions in any organs described above was evaluated as 0.

### 2.5. Single-Cell Isolation from Lungs, Spleen, and Blood

Single mononuclear cell suspensions from the lungs and spleen were prepared following the protocol described elsewhere [5]. Blood samples diluted with the same volume of PBS were overlaid onto Histopaque1077 and centrifuged at 560× *g* for 20 min. Mononuclear cells aspirated from the interface were washed 3 times with RPMI 1640 (Millipore-Sigma) with 100 U/mL of penicillin and 100 μg/mL of streptomycin. Cells from the lungs, spleen, and blood were resuspended in complete RPMI cell culture medium: the RPMI 1640 medium was supplemented with 10% of fetal bovine serum, 100 U/mL of penicillin and 100 μg/mL of streptomycin, 50 μg/mL of Gentamicin (Gibco), 25 mM of HEPES (Gibco), and 71.5 μM of 2-mercaptoethanol (Millipore-Sigma). Live cell numbers were calculated using a haemocytometer and Trypan blue. Five hundred thousand mononuclear cells were used for surface molecular and mTGF-β staining following the protocol described elsewhere [5]. For the purpose of IFN-γ and TGF-β staining, 1 × 10^6^ cells were cultured with Golgi Plug and DNase I in the presence or absence of 20 ng/mL of phorbol 12-myristate 13-acetate (PMA) (Millipore-Sigma) and 500 ng/mL of ionomycin (ION) (Millipore-Sigma) in 96-well round bottom plates for 4 h at 41 °C, 5% CO_2_. The protocol of the degranulation assay is described elsewhere [5]. 

### 2.6. Flow Cytometry

The protocols for surface molecule and mTGF-β staining, intracellular cytokine staining, and CD107a staining are described elsewhere [5]. Antibodies used in the experiment are listed in Table 1. Stained cells were acquired on a FACSCanto II flow cytometer (Beckton Dickinson Biosciences). FlowJo version 10.8.1 (TreeStar Inc., Ashland, OR, USA) was used to analyze the acquired data. Single cells were examined by the side scatter area (SSC-A) versus forward scatter area (FSC-A), followed by the forward scatter height (FSC-H) versus FSC-A. Dead cells were excluded by 7-AAD (Invitrogen) staining. Representative gating strategies are shown in Supplementary Figure S1. Fluorescence minus one control were used to identify IFN-γ, TGF-β, CD25, and degranulation (CD107a) activity.

### 2.7. DNA Extraction

DNA was extracted from feather tips using Trizol reagent according to the instruction provided by the manufacturer. NanoDrop^®^ ND-1000 spectrophotometry (Thermo Fisher Scientific Inc.) was used to calculate the DNA concentration.

### 2.8. RNA Extraction and cDNA Synthesis

The spleen, lungs, and PBMCs were homogenized in 1 mL of Trizol reagent and RNA was extracted according to the instruction provided by the manufacturer. The RNA concentration was calculated by NanoDrop^®^ ND-1000 spectrophotometry. cDNA was synthesized from DNase-treated RNA (spleen and lung samples: 1 μg of RNA, PBMC samples: 500 ng of RNA) using Oligo (dT) 12–18 primers and the SuperScript™ II Reverse Transcriptase (Invitrogen) according to the manufacturer’s instruction. Diluted cDNA at 1:10 (spleen and lung samples) or 1:5 (PBMC samples) in nuclease-free water was used in a real-time PCR to evaluate virus or host gene expressions.

### 2.9. Real-Time Quantitative Polymerase Chain Reaction (RT-qPCR)

To determine MDV genome copy numbers in 100 ng of DNA extracted from feathers, the viral meq gene was quantified using the primer set (Table 2) as described previously [22]. An RT-qPCR was conducted using the LightCycler^®^ 480 II instrument (Roche Diagnostics GmbH, Mannheim, Germany) and LightCycler^®^ 480 SYBR Green I Master Mix (Roche Diagnostics). Primer sequences of chicken or MDV genes and cycling parameters are provided in Table 2 and Table 3, respectively. The primers were synthesized by Thermo Fisher Scientific Inc. The relative expression of target genes was calculated using LightCycler^®^ 480 advanced relative quantification software in relation to chicken β-actin. Serial dilution of the standard plasmid for meq was prepared for the quantification of meq gene expression by LightCycler^®^ 480 quantification software (Roche Diagnostics).

### 2.10. Statistical Analysis

A statistical analysis of the ex vivo experimental data was performed with Levene’s test for equality of variances followed by a two-tailed t-test or the Mann–Whitney U test. A statistical analysis of the tumor incidence data was performed with Fisher’s exact test. A statistical analysis of the tumor scoring and the in vivo experimental data was performed with Levene’s test for equality of variances, followed by a one-way analysis of variance (ANOVA), Tukey’s post hoc test or Kruskal–Wallis test, and Dunn’s test with Benjamini and Hochberg adjustment. All the statistical analyses were performed using IBM SPSS statistics 28 (IBM, Armonk, New York, USA) or GraphPad Prism 7 (GraphPad Software Inc., La Jolla, CA, USA). The data are represented as mean ± standard error (SE).

## 3. Results

### 3.1. TCRγδ Stimulation Induced IFN-γ Production by Chicken γδ T Cells

**Figure 1:**
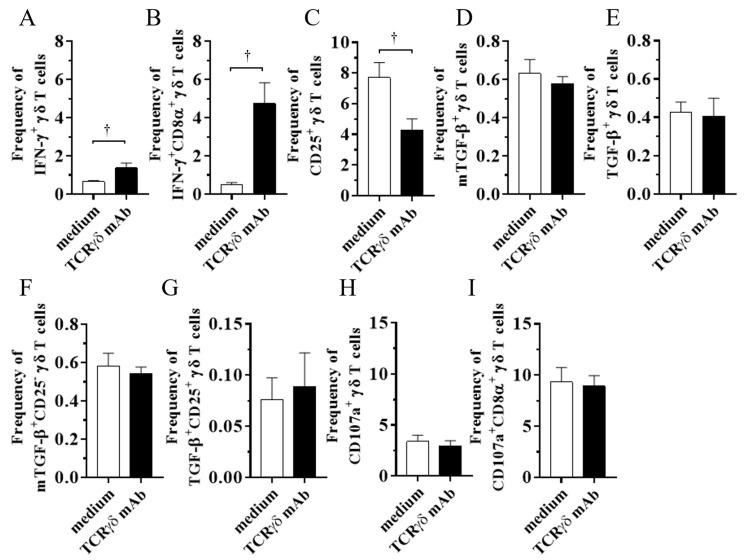
Cytokine and CD107a profiles in γδ T cells at 4 or 18 h post-stimulation with anti-TCRγδ antibody. The PBMCs obtained from healthy SPF chickens were stimulated with or without anti-TCRγδ antibody, and degranulation activity of γδ T cells (4 h post-stimulation (hps)) and cytokine production (18 hps) were analyzed using flow cytometry. Percentages of IFN-γ^+^ γδ T cells within γδ T cell populations (**A**), IFN-γ^+^CD8α^+^ γδ T cells within CD8α^+^ γδ T cell populations (**B**), CD25^+^ γδ T cells within γδ T cell populations (**C**), mTGF-β γδ T cells within γδ T cell populations (**D**), TGF-β γδ T cells within γδ T cell populations (**E**), mTGF-β^+^CD25^-^ γδ T cells within γδ T cell populations (**F**), TGF-β^+^CD25^+^ γδ T cells within γδ T cell populations (**G**), CD107a^+^ γδ T cells within γδ T cell populations (**H**), and CD107a^+^CD8α^+^ γδ T cells within CD8α^+^ γδ T cell populations (**I**) derived from the PBMCs are shown. TCRγδ-unstimulated cells were used as a negative control. Data represent the mean of 6 biological replicates ± SE. Significant differences are indicated by †: *p* < 0.05.

To determine if TCRγδ stimulation via their TCR receptors induces cytokine production and degranulation by chicken γδ T cells, PBMCs were stimulated with 10 μg/mL of anti-chicken TCRγδ mAb, and then intracellular IFN-γ and TGF-β cytokine production as well as mTGF-β, CD25, and CD107a expression levels were analyzed using flow cytometry. Since our previous study suggested the different TGF-β expression pattern between a soluble form and a membrane-bound form in chicken γδ T cells, both the mTGF-β expression and intracellular TGF-β production by γδ T cells were evaluated [5]. Data were presented as the percentage of IFN-γ^+^/ mTGF-β^+^/ TGF-β^+^/ CD25^+^/ TGF-β^+^CD25^-^/ TGF-β^+^CD25^+^/ CD107a^+^ γδ T cells within γδ T cell populations and IFN-γ^+^CD8α^+^/ CD107a^+^CD8α^+^ γδ T cells within CD8α^+^ γδ T cell populations. TCRγδ stimulation induced significant IFN-γ production by γδ T cells (*p* < 0.05) and CD8α^+^ γδ T cells (*p* < 0.05) (Figure 1A,B), whereas the CD25 expression on TCRγδ-stimulated γδ T cells was lower than TCRγδ-unstimulated γδ T cells (*p* < 0.05) (Figure 1C). Significant differences between the TCRγδ-stimulated and the unstimulated conditions were not observed in the frequency of mTGF-β γδ T cells, TGF-β^+^ γδ T cells, TGF-β^+^CD25^-^ γδ T cells, TGF-β^+^CD25^+^ γδ T cells, CD107a^+^ γδ T cells, and CD107a^+^CD8α^+^ γδ T cells (*p* ≥ 0.05) (Figure 1D–I). At 18 hps, the mean percentage of γδ T cells and αβ T cells within the live lymphocyte population was 10.83 ± 0.93% and 49.77 ± 4.58%, respectively, in the TCRγδ-unstimulated condition and 9.95 ± 1.27% and 53.28 ± 5.03%, respectively, in the TCRγδ-stimulated condition. There were no significance differences in T cell populations between in TCRγδ-unstimulated PBMCs and TCRγδ-stimulated PBMCs.

### 3.2. TCRγδ Stimulation Induced Upregulation of IFN-γ, IL-2, and IL-17 Gene Expression and Downregulation of TGF-β Gene Expression in the PBMCs

**Figure 2:**
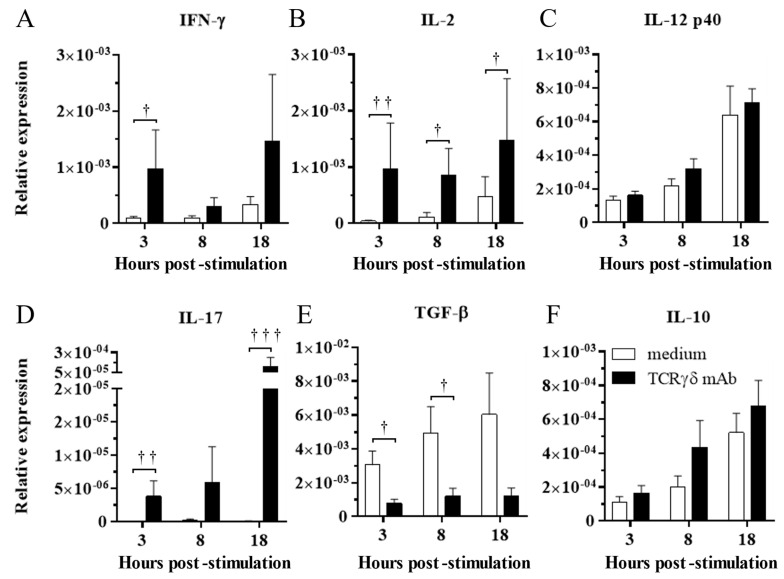
Cytokine expression profiles in the PBMCs stimulated with or without anti-TCRγδ antibody at 3, 8, and 18 hps. The PBMCs obtained from healthy SPF chickens were stimulated with or without anti-TCRγδ antibody for 3, 8, and 18 h, and RNA were extracted from the cultured PBMCs. Expression of cytokines, IFN-γ (**A**), IL-2 (**B**), IL-12p40 (**C**), IL-17A (**D**), TGF-β (**E**), and IL-10 (**F**) was determined by real-time PCR. Target genes were normalized by β-actin. TCRγδ-unstimulated cells were used as a negative control. Data represent the mean of 6 biological replicates ± SE at each time point. Significant differences are indicated by †: *p* < 0.05, ††: *p* < 0.01, and †††: *p* < 0.001.

To determine the effect of TCRγδ stimulation, the PBMCs were stimulated with 10 μg/mL of anti-chicken TCRγδ mAb, and IFN-γ, IL-2, IL-12p40, IL-17A, TGF-β, and IL-10 gene expression in the stimulated PBMCs was analyzed at 3, 8, and 18 hps by real-time PCR. TCRγδ stimulation induced the significant upregulation of IFN-γ and IL-2 gene expression in the PBMCs at 3 hps (*p* < 0.05 and *p* < 0.01, respectively) (Figure 2A,B). The gene expression of IL-2 in stimulated PBMCs was significantly higher at 8 and 18 hps compared to unstimulated PBMCs (*p* < 0.05) (Figure 2B). There were no significant differences in IL-12p40 gene expression between stimulated PBMCs and unstimulated PBMCs (*p* ≥ 0.05) (Figure 2C). The significantly higher gene expression of IL-17A was observed in stimulated PBMCs than in unstimulated PBMCs at 3 and 18 hps (*p* < 0.01 and *p* < 0.001, respectively) (Figure 2D). TCRγδ stimulation induced the significant downregulation of TGF-β gene expression in the PBMCs at 3 and 8 hps (*p* < 0.05), whereas significant differences in IL-10 gene expression were not observed between the stimulated PBMCs and unstimulated PBMCs (Figure 2E,F).

### 3.3. Adoptive Transfer of TCRγδ-Activated PBMCs Reduced Tumor Incidence and Suppressed Viral Replication in MDV-Challenged Chickens

**Figure 3:**
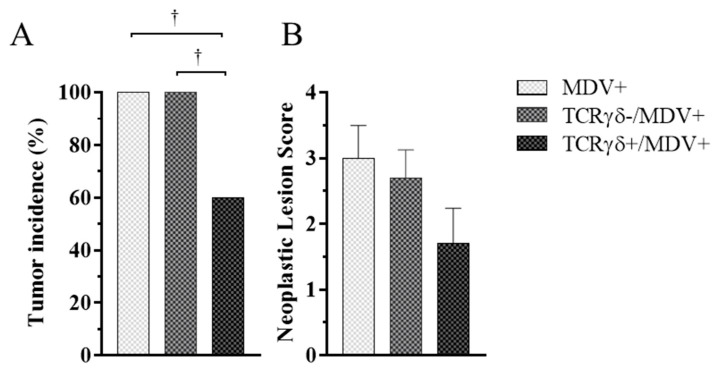
Tumor incidence and neoplastic lesion scores at 21 days post-cell infusion and MDV challenge. TCRγδ-activated or -unactivated PBMCs were infused to recipient chickens at 21-days-old, and the chickens were challenged with MDV. Tumor incidence (**A**) and neoplastic lesion scores (**B**) were determined by necropsy in the MDV+, the TCRγδ-/MDV+, and the TCRγδ+/MDV+ groups at 21 dpi. Tumor scores were assessed by the number of tumor-bearing organs. Data of the neoplastic lesion scores represent the mean of 10 biological replicates ± SE in each group. Significant differences are indicated by †: *p* < 0.05.

To investigate whether activated γδ T cells are involved in the prevention of MD-lymphoma, chickens received TCRγδ-activated PBMCs, followed by the MDV challenge. Tumor incidence and neoplastic lesion scores were assessed at 21 dpi. Chickens in the TCRγδ+/MDV+ group showed significantly lower tumor incidence compared to the other groups (*p* < 0.05) (Figure 3A). However, there were no significant differences between groups in neoplastic lesion scores (*p* ≥ 0.05) (Figure 3B).

**Figure 4:**
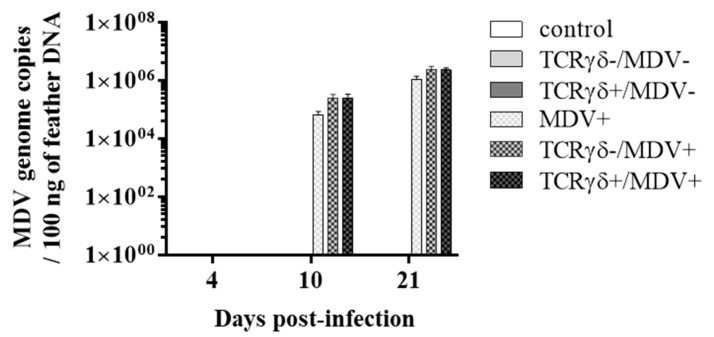
MDV meq gene copy number from feather tips at 4, 10, and 21 dpi. TCRγδ-activated or -unactivated PBMCs were infused to recipient chickens at 21-days-old, and the chickens were challenged with MDV. Feather samples were collected from the control, the TCRγδ-/MDV-, the TCRγδ+/MDV-, the MDV+, the TCRγδ-/MDV+, and the TCRγδ+/MDV+ groups at 4, 10, and 21 dpi. Meq gene was evaluated in 100 ng of DNA extracted from feathers. Data represent the mean of 5 biological replicates ± SE in each group at each time point.

**Figure 5:**
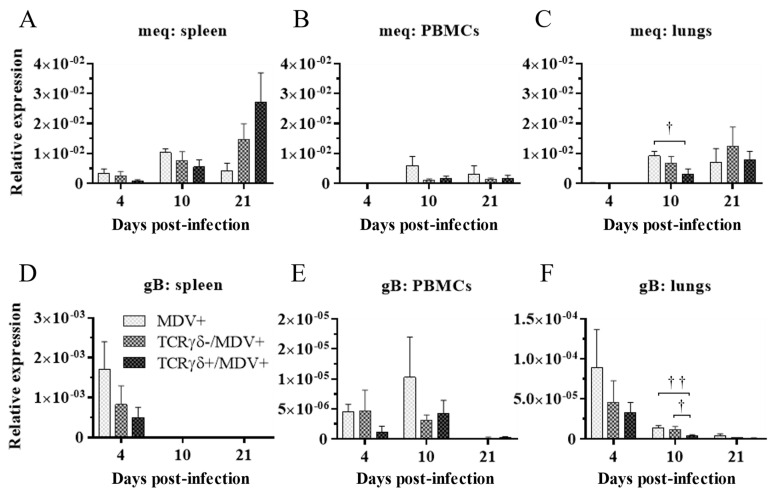
Transcripts of MDV genes in the spleen, PBMCs, and lungs at 4, 10, and 21 dpi. TCRγδ-activated or -unactivated PBMCs were infused to recipient chickens at 21-days-old, and the chickens were challenged with MDV. Spleen, PBMCs, and lungs samples were collected from the MDV+, the TCRγδ-/MDV+, and the TCRγδ+/MDV+ groups at 4, 10, and 21 dpi. Transcripts of MDV genes, meq (**A**–**C**), and gB (**D**–**F**), in the spleen, PBMCs, and lungs collected from each group at 4, 10, and 21 dpi were determined by real-time PCR. Target genes were normalized by β-actin. Data represent the mean of 5 biological replicates ± SE in each group at each time point. Significant differences are indicated by †: *p* < 0.05 and ††: *p* < 0.01.

Next, we examined if the infusion of γδ T cells regulates virus production in feathers or virus replication in MDV-challenged chickens. To this end, the MDV genome load in feathers and transcripts of viral genes, meq, and glycoprotein B (gB), in the spleen, PBMCs, and lungs were analyzed. Significant differences in the MDV genome load were not observed in feathers between groups (*p* ≥ 0.05) (Figure 4). Significant differences in meq and gB gene expression were not observed in the spleen or PBMCs between groups (*p* ≥ 0.05) (Figure 5A,B,D,E). However, the gene expression of meq was significantly lower in the lungs of the TCRγδ+/MDV+ group at 10 dpi than in the MDV+ group (*p* < 0.05) (Figure 5C). Lower gene expression of gB in the lungs was observed in the TCRγδ+/MDV+ group compared to the MDV+ group (*p* < 0.01) and the TCRγδ-/MDV+ group (*p* < 0.05) (Figure 5F).

### 3.4. Lower Frequency of γδ T Cells in the Spleen of the TCRγδ-/MDV+ Group at 10 dpi and Higher Frequency of αβ T Cells in the Spleen and PBMCs of the TCRγδ+/MDV+ Group at 21 dpi

**Figure 6:**
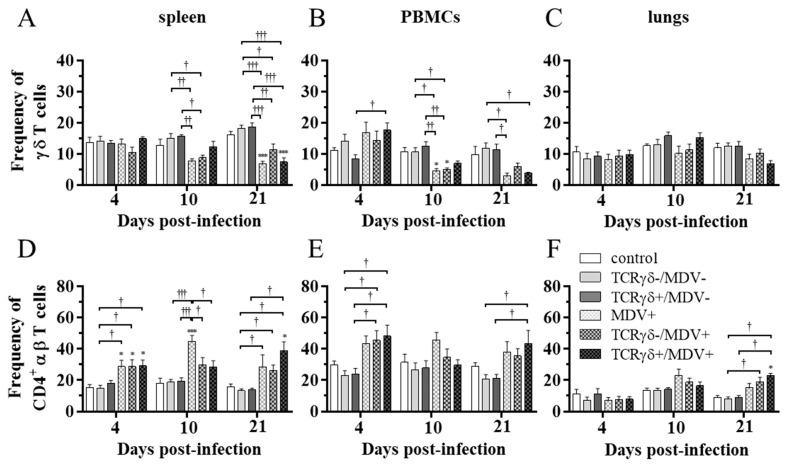
Frequency of γδ T cells and CD4^+^ αβ T cells in the spleen, PBMCs, and lungs, at 4, 10, and 21 dpi. TCRγδ-activated or -unactivated PBMCs were infused to recipient chickens at 21-days-old, and the chickens were challenged with MDV. Percentages of γδ T cells (**A**–**C**) and CD4^+^ αβ T cells (**D**–**F**) within live lymphocyte populations in the spleen, PBMCs, and lungs collected from the control, the TCRγδ-/MDV-, the TCRγδ+/MDV-, the MDV+, the TCRγδ-/MDV+, and the TCRγδ+/MDV+ groups at 4, 10, and 21 dpi are shown. Data represent the mean of 5 biological replicates ± SE in each group at each time point. Significant differences are indicated by *: *p* < 0.05 (vs. control), ***: *p* < 0.001 (vs. control), †: *p* < 0.05, ††: *p* < 0.01, and †††: *p* < 0.001.

To examine how the infusion of TCRγδ-activated PBMCs affects T cell frequencies in MDV-challenged chickens, the frequency of γδ T cells and CD4^+^ αβ T cells in the spleen, PBMCs, and lungs at 4, 10, and 21 dpi was analyzed using flow cytometry. Data are presented as the percentage of γδ T cells or CD4^+^ αβ T cells within live lymphocyte populations. A significant decrease in γδ T cell frequency in the spleen and PBMCs was observed in the TCRγδ-/MDV+ group compared to the TCRγδ-/MDV- group at 10 dpi (*p* < 0.05), but not in the TCRγδ+/MDV+ group compared to the TCRγδ+/MDV- group (*p* ≥ 0.05) (Figure 6A,B). There were no significant differences in the frequency of γδ T cells in the lungs (*p* ≥ 0.05) (Figure 6C). The frequency of CD4^+^ αβ T cells in the spleen, PBMCs, and lungs was significantly higher in the TCRγδ+/MDV+ group than that in the TCRγδ+/MDV- group at 21 dpi (*p* < 0.05) (Figure 6D–F).

### 3.5. Circulating γδ T Cells in the TCRγδ+/MDV+ Group Showed a Higher Degranulation Activity at 10 and 21 dpi

**Figure 7:**
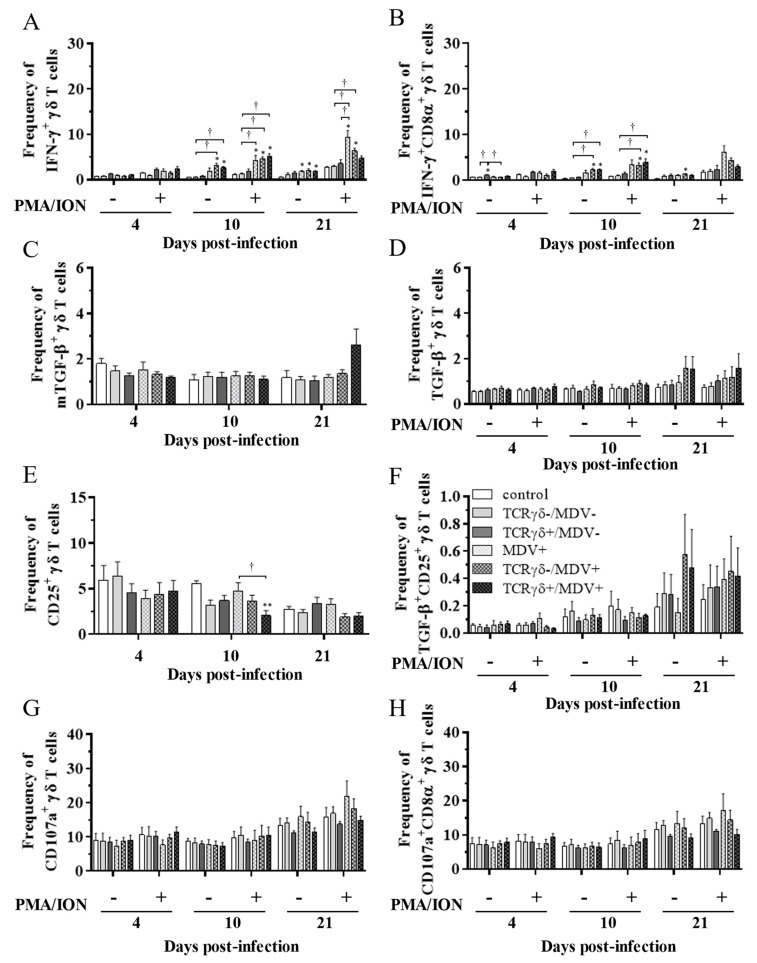
Cytokine and immune system molecule profiles in γδ T cells derived from the spleen at 4, 10, and 21 dpi. Mononuclear cells isolated from the spleen of the control, the TCRγδ-/MDV-, the TCRγδ+/MDV-, the MDV+, the TCRγδ-/MDV+, and the TCRγδ+/MDV+ groups were cultured with and without PMA/ION for 4 h. Percentages of IFN-γ^+^ γδ T cells (**A**), IFN-γ^+^CD8α^+^ γδ T cells (**B**), mTGF-β γδ T cells (**C**), TGF-β γδ T cells (**D**), CD25^+^ γδ T cells (**E**), TGF-β^+^CD25^+^ γδ T cells (**F**), CD107a^+^ γδ T cells (**G**), and CD107a^+^CD8α^+^ γδ T cells (**H**) within γδ T cell populations in the spleen collected from each group at 4, 10, and 21 dpi are shown. Data represent the mean of 5 biological replicates ± SE in each group at each time point. Significant differences are indicated by *: *p* < 0.05 (vs. control), **: *p* < 0.01 (vs. control) and †: *p* < 0.05.

**Figure 8:**
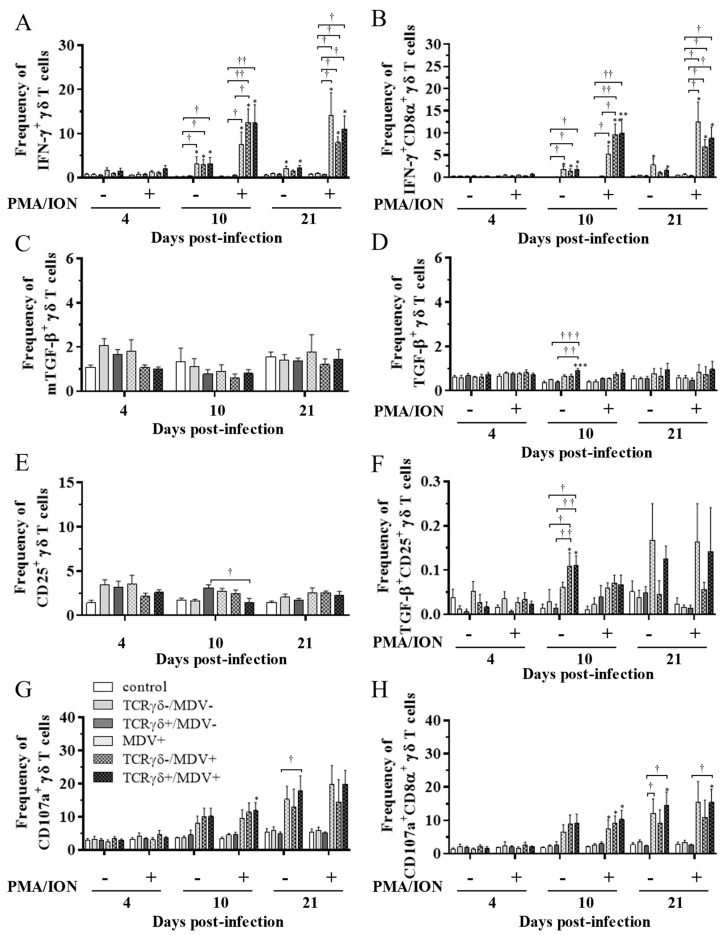
Cytokine and immune system molecule profiles in γδ T cells derived from the PBMCs at 4, 10, and 21 dpi. The PBMCs obtained from the control, the unstimulated PBMCs control, the TCRγδ-/MDV-, the TCRγδ+/MDV-, the MDV+, the TCRγδ-/MDV+, and the TCRγδ+/MDV+ groups were cultured with and without PMA/ION for 4 h. Percentages of IFN-γ+ γδ T cells (**A**), IFN-γ+CD8α+ γδ T cells (**B**), mTGF-β γδ T cells (**C**), TGF-β γδ T cells (**D**), CD25+ γδ T cells (**E**), TGF-β+CD25+ γδ T cells (**F**), CD107a+ γδ T cells (**G**), and CD107a+CD8α+ γδ T cells (**H**) within γδ T cell populations in the PBMCs collected from each group at 4, 10, and 21 dpi are shown. Data represent the mean of 5 biological replicates ± SE in each group at each time point. Significant differences are indicated by *: *p* < 0.05 (vs. control), **: *p* < 0.01 (vs. control), ***: *p* < 0.001 (vs. control), †: *p* < 0.05, ††: *p* < 0.01, and †††: *p* < 0.001.

**Figure 9:**
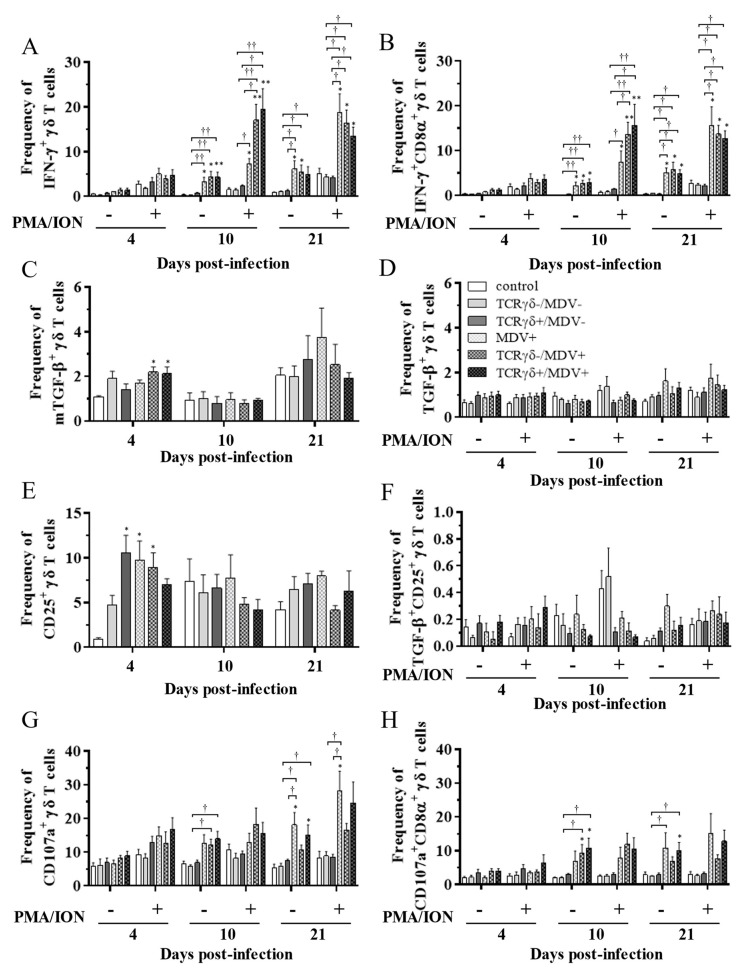
Cytokine and immune system molecule profiles in γδ T cells derived from the lungs at 4, 10, and 21 dpi. Mononuclear cells isolated from the lungs of the control, the TCRγδ-/MDV-, the TCRγδ+/MDV-, the MDV+, the TCRγδ-/MDV+, and the TCRγδ+/MDV+ groups were cultured with and without PMA/ION for 4 h. Percentages of IFN-γ^+^ γδ T cells (**A**), IFN-γ^+^CD8α^+^ γδ T cells (**B**), mTGF-β γδ T cells (**C**), TGF-β γδ T cells (**D**), CD25^+^ γδ T cells (**E**), TGF-β^+^CD25^+^ γδ T cells (**F**), CD107a^+^ γδ T cells (**G**), and CD107a^+^CD8α^+^ γδ T cells (**H**) within γδ T cell populations in the lungs collected from each group at 4, 10, and 21 dpi are shown. Data represent the mean of 5 biological replicates ± SE in each group at each time point. Significant differences are indicated by *: *p* < 0.05 (vs. control), **: *p* < 0.01 (vs. control), †: *p* < 0.05, and ††: *p* < 0.01.

To examine the kinetics of γδ T cells in the TCRγδ+/MDV+ group, γδ T cells derived from the spleen (Figure 7), PBMCs (Figure 8), or lungs (Figure 8) were cultured in the presence of phorbol 12-myristate 13-acetate and ionomycin (PMA/ION) (potential activity) or the absence of PMA/ION (baseline activity). IFN-γ and TGF-β production as well as CD25 and CD107a expression levels in γδ T cells were analyzed using flow cytometry at 4, 10, and 21 dpi. Data are presented as the percentage of IFN-γ^+^/ IFN-γ^+^CD8α^+^/ mTGF-β^+^/ TGF-β^+^/ CD25^+^/ TGF-β^+^CD25^+^/ CD107a^+^/ CD107a^+^CD8α^+^ γδ T cells within γδ T cell populations. The frequency of IFN-γ^+^ γδ T cells was significantly increased in the spleen of both the TCRγδ-/MDV+ and the TCRγδ+/MDV+ groups compared to the control group at 10 dpi (*p* < 0.05) without PMA/ION re-stimulation, which was also observed in the PMA/ION-re-stimulated condition (*p* < 0.05) (Figure 7A). A significantly higher frequency of IFN-γ^+^CD8α^+^ γδ T cells was observed in the spleen of the TCRγδ+/MDV+ group and the TCRγδ-/MDV+ group at 10 dpi under the PMA/ION-re-stimulated condition compared to the control group (*p* < 0.05) (Figure 7B). No significant differences in the frequency of mTGF-β^+^ γδ T cells, TGF-β^+^ γδ T cells, and TGF-β^+^CD25^+^ γδ T cells were observed in the spleen between groups (*p* ≥ 0.05) (Figure 7C,D,F). The expression of CD25 on γδ T cells in the spleen of the TCRγδ+/MDV+ group at 10 dpi was significantly lower than that in the control group (*p* < 0.01) and in the MDV+ group (*p* < 0.05) (Figure 7E). Significant differences were not observed between groups in the frequency of CD107a^+^ γδ T cells and CD107a^+^CD8α^+^ γδ T cells (*p* ≥ 0.05) (Figure 7G,H). The frequency of IFN-γ^+^ γδ T cells was significantly increased in the PBMCs of chickens from the TCRγδ-/MDV+ and the TCRγδ+/MDV+ groups at 10 dpi in both PMA/ION-unstimulated and -re-stimulated conditions compared to the control group (*p* < 0.05) (Figure 8A). Higher IFN-γ production was observed in circulating CD8α^+^ γδ T cells of chickens in the TCRγδ-/MDV+ and the TCRγδ+/MDV+ groups upon PMA/ION re-stimulation at 10 dpi compared to the control group (*p* < 0.01) (Figure 8B). There was no significant difference between groups in the frequency of mTGF-β^+^ γδ T cells (*p* ≥ 0.05) (Figure 8C). TGF-β production was significantly increased in the circulating γδ T cells of TCRγδ+/MDV+ chickens at 10 dpi under the PMA/ION-unstimulated condition compared to the control group (*p* < 0.001) and the TCRγδ+/MDV- group (*p* < 0.01) (Figure 8D). The CD25 expression on the circulating γδ T cells in the TCRγδ+/MDV+ group at 10 dpi was significantly lower than that in the TCRγδ+/MDV- group (*p* < 0.05) (Figure 8E). Chickens in both the TCRγδ+/MDV+ and the TCRγδ-/MDV+ groups had a significantly higher frequency of TGF-β^+^CD25^+^ γδ T cells in the PBMCs than that in the control group without PMA/ION re-stimulation (*p* < 0.05) (Figure 8F). Circulating γδ T cells and CD8α^+^ γδ T cells in the TCRγδ+/MDV+ group had a significantly higher degranulation activity upon re-stimulation with PMA/ION at 10 dpi compared to the control group (*p* < 0.05) (Figure 8G,H). A significantly higher frequency of CD107a^+^CD8α^+^ γδ T cells derived from the PBMCs of the TCRγδ+/MDV+ group was observed than that of the control group and the TCRγδ+/MDV- group at 21 dpi upon re-stimulation with PMA/ION (*p* < 0.05) and without PMA/ION re-stimulation (*p* < 0.05) (Figure 8H). In the lungs, chickens in the TCRγδ+/MDV+ group showed a significantly higher frequency of IFN-γ^+^ γδ T cells and IFN-γ^+^CD8α^+^ γδ T cells than that in the control group upon PMA/ION re-stimulation at 10 dpi (*p* < 0.01), which was observed in the TCRγδ-/MDV+ group as well (*p* < 0.01) (Figure 9A,B). A significantly higher frequency of IFN-γ^+^CD8α^+^ γδ T cells was observed in all the MDV-challenged groups than that in the control group at 21 dpi with and without PMA/ION re-stimulation (*p* < 0.05) (Figure 9B). The frequency of mTGF-β^+^ γδ T cells in lungs was higher in the TCRγδ+/MDV+ and the TCRγδ-/MDV+ groups than that in the control group at 4 dpi (*p* < 0.05), but significant differences between groups were not observed in the frequency of TGF-β^+^ γδ T cells (*p* ≥ 0.05) and TGF-β^+^CD25^+^ γδ T cells (*p* ≥ 0.05) (Figure 9C,D,F). The expression of CD25 on γδ T cells in the lungs of the MDV+ group and the TCRγδ-/MDV+ group at 4 dpi was significantly upregulated compared to the control group (*p* < 0.05), while there was no significant difference between the TCRγδ+/MDV+ group and the control group (*p* ≥ 0.05) (Figure 9E). A significantly higher CD107a expression was observed in CD8α^+^ γδ T cells derived from the lungs of the TCRγδ-/MDV+ and the TCRγδ+/MDV+ group than that in the control group at 10 dpi under the PMA/ION-unstimulated condition (*p* < 0.05), and in γδ T cells and CD8α^+^ γδ T cells derived from the lungs of the TCRγδ+/MDV+ group compared to the control group at 21 dpi under the PMA/ION-unstimulated condition (*p* < 0.05) (Figure 9G,H).

### 3.6. Infusion of TCRγδ-Activated PBMCs Induced IFN-γ Gene Expression in the Spleen at 4, 10, and 21 dpi and Granzyme A Gene Expression at 10 dpi

**Figure 10:**
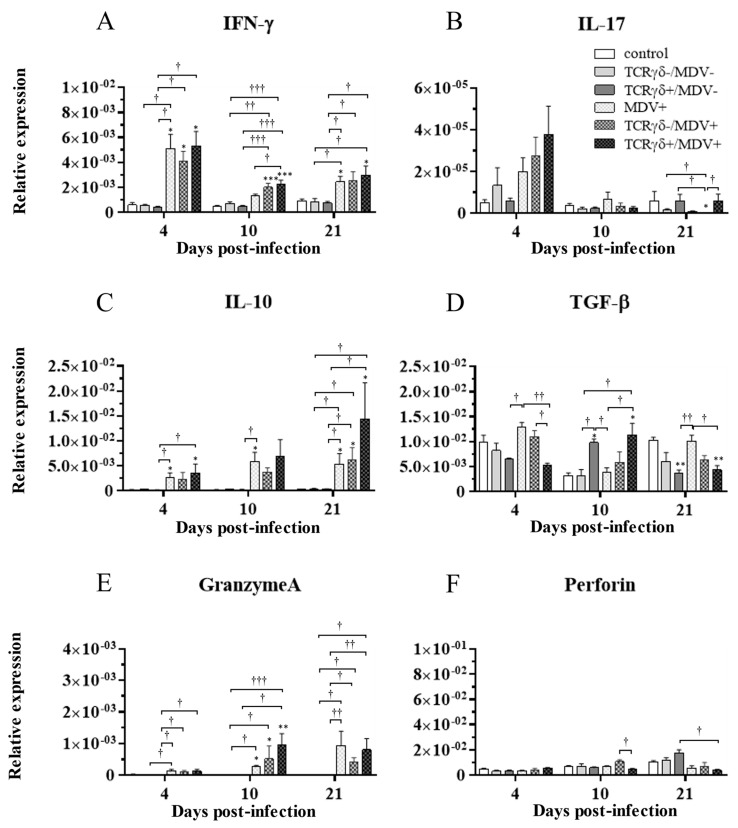
Cytokine, granzyme A, and perforin gene expression profiles in the spleen at 4, 10, and 21 dpi. Gene expression of IFN-γ (**A**), IL-17A (**B**), IL-10 (**C**), TGF-β (**D**), granzyme A (**E**), and perforin (**F**) in the spleen collected from the control, the unstimulated PBMCs control, the TCRγδ-/MDV-, the TCRγδ+/MDV-, the MDV+, the TCRγδ-/MDV+, and the TCRγδ+/MDV+ groups at 4, 10, and 21 dpi was determined by real-time PCR. Target genes were normalized by β-actin. Data represent the mean of 5 biological replicates ± SE in each group at each time point. Significant differences are indicated by *: *p* < 0.05 (vs. control), **: *p* < 0.01 (vs. control), ***: *p* < 0.001 (vs. control), †: *p* < 0.05, ††: *p* < 0.01, and †††: *p* < 0.001.

**Figure 11:**
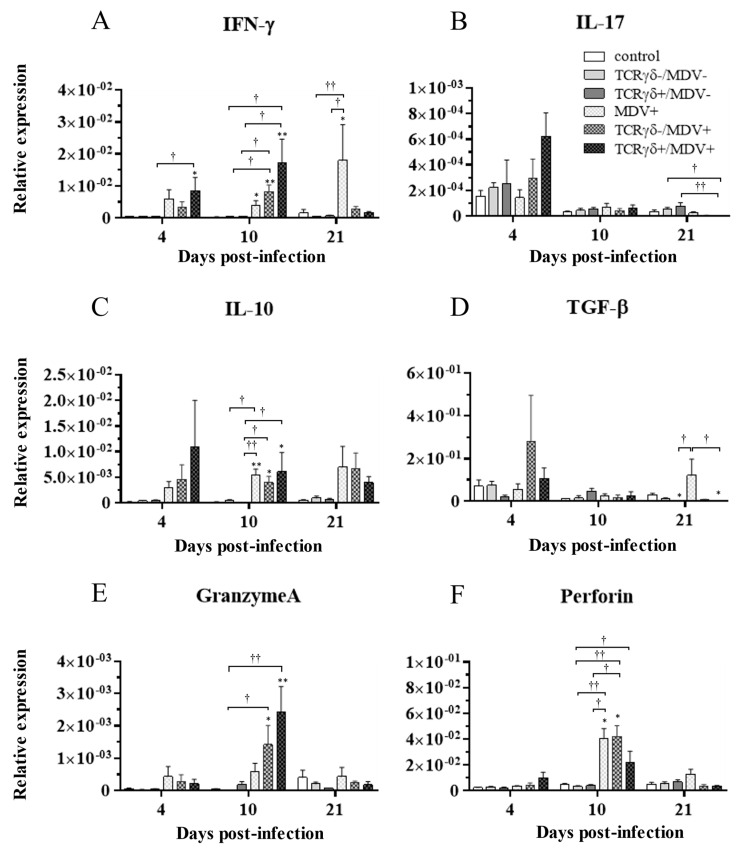
Cytokine, granzyme A, and perforin gene expression profiles in the PBMCs at 4, 10, and 21 dpi. Gene expression of IFN-γ (**A**), IL-17A (**B**), IL-10 (**C**), TGF-β (**D**), granzyme A (**E**), and perforin (**F**) in the PBMCs collected from the control, the unstimulated PBMCs control, the TCRγδ-/MDV-, the TCRγδ+/MDV-, the MDV+, the TCRγδ-/MDV+, and the TCRγδ+/MDV+ groups at 4, 10, and 21 dpi was determined by real-time PCR. Target genes were normalized by β-actin. Data represent the mean of 5 biological replicates ± SE in each group at each time point. Significant differences are indicated by *: *p* < 0.05 (vs. control), **: *p* < 0.01 (vs. control), †: *p* < 0.05, and ††: *p* < 0.01.

**Figure 12:**
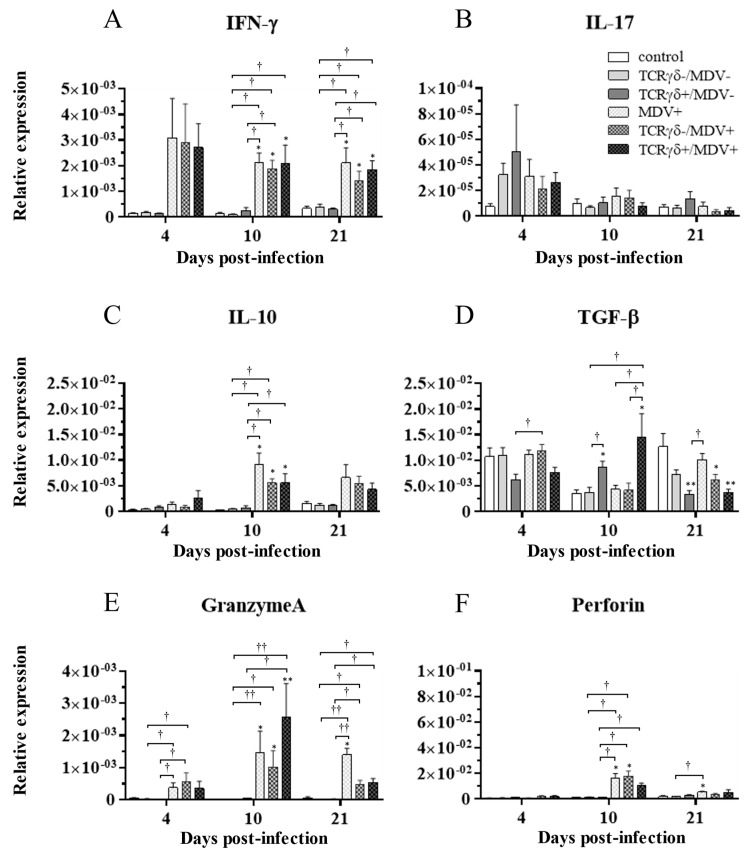
Cytokine, granzyme A, and perforin gene expression profiles in the lungs at 4, 10, and 21 dpi. Gene expression of IFN-γ (**A**), IL-17A (**B**), IL-10 (**C**), TGF-β (**D**), granzyme A (**E**), and perforin (**F**) in the lungs collected from the control, TCRγδ-/MDV-, the TCRγδ+/MDV-, the MDV+, the TCRγδ-/MDV+, and the TCRγδ+/MDV+ groups at 4, 10, and 21 dpi was determined by real-time PCR. Target genes were normalized by β-actin. Data represent the mean of 5 biological replicates ± SE in each group at each time point. Significant differences are indicated by *: *p* < 0.05 (vs. control), **: *p* < 0.01 (vs. control), †: *p* < 0.05, and ††: *p* < 0.01.

To examine how the infusion of TCRγδ-activated PBMCs alters cytokine and molecules related to cytotoxic activity in MDV-challenged chickens, the gene expression of IFN-γ, IL-17A, IL-10, TGF-β, granzyme A, and perforin in the spleen (Figure 10), PBMCs (Figure 11), and lungs (Figure 12) was analyzed using a real-time PCR. The gene expression of IFN-γ in the spleen of the TCRγδ+/MDV+ group was significantly higher than that of the TCRγδ+/MDV- at 4, 10, and 21 dpi (*p* < 0.05, *p* < 0.001, and *p* < 0.05, respectively) (Figure 10A). At 10 dpi, IFN-γ gene expression was significantly upregulated in the spleen of the TCRγδ+/MDV+ group compared to the control group and the MDV+ group (*p* < 0.001 and *p* < 0.05, respectively) (Figure 10A). Chickens in the TCRγδ+/MDV+ group showed higher IL-17A gene expression in the spleen at 21 dpi compared to the TCRγδ-/MDV+ group (*p* < 0.05) (Figure 10B). The gene expression of IL-10 in the spleen of the TCRγδ+/MDV+ group was significantly increased at 4 and 21 dpi compared to that of the control group (*p* < 0.05) and the TCRγδ+/MDV- group (*p* < 0.05) (Figure 10C). Significantly higher TGF-β gene expression was observed in the spleen of the TCRγδ+/MDV- and the TCRγδ+/MDV+ group at 10 dpi compared to the control group (*p* < 0.05), while TGF-β gene expression in the spleen of the TCRγδ+/MDV+ group was lower than that in the MDV+ group at 4 and 21 dpi (*p* < 0.01 and *p* < 0.05, respectively), in the TCRγδ-/MDV+ group at 4 dpi (*p* < 0.05), and in the control group at 21 dpi (*p* < 0.01) (Figure 10D). Granzyme A gene expression was significantly upregulated in the spleen of the TCRγδ+/MDV+ group at 10 dpi compared to that in the control group (*p* < 0.01), which was also observed in the PBMCs and lungs (*p* < 0.01) (Figure 10E, Figure 11E and Figure 12E). The significantly lower gene expression of perforin in the spleen was observed in the TCRγδ+/MDV+ group compared to the TCRγδ-/MDV+ group at 10 dpi (*p* < 0.05) and the TCRγδ+/MDV- group at 21 dpi (*p* < 0.05) (Figure 10F). Similar to the results observed in the spleen, chickens in the TCRγδ+/MDV+ group showed a significantly higher IFN-γ gene expression in the PBMCs at 4 dpi compared to the control group and the TCRγδ+/MDV- group (*p* < 0.05) (Figure 11A). The IFN-γ gene expression in the PBMCs of the TCRγδ+/MDV+ group and the TCRγδ-/MDV+ group was higher than that of the control group at 10 dpi (*p* < 0.01). The expression of the IL-17A gene in the PBMCs of the TCRγδ+/MDV+ group was significantly lower than that of the TCRγδ+/MDV- group at 21 dpi (*p* < 0.01) (Figure 11B). IL-10 gene expression in the PBMCs of the TCRγδ+/MDV+ group was higher than that in the control group and the TCRγδ+/MDV- group at 10 dpi (*p* < 0.05) (Figure 11C). Chickens in the TCRγδ+/MDV+ group showed significantly lower TGF-β gene expression in the PBMCs compared to the control group and the MDV+ group at 21 dpi (*p* < 0.05) (Figure 11D). Perforin gene expression was also upregulated in the MDV+ group and the TCRγδ-/MDV+ group at 10 dpi compared to the control group (*p* < 0.05) (Figure 11F). In the lungs, all the MDV-challenged groups showed significantly higher IFN-γ gene expression compared to the control groups at 10 and 21 dpi (*p* < 0.05) (Figure 12A). There were no significant differences between groups in the expression of the IL-17A gene in the lungs (*p* ≥ 0.05) (Figure 12B). IL-10 gene expression in the lungs was significantly higher in all the MDV-challenged groups than that in the control group at 10 dpi (*p* < 0.05) (Figure 12C). The expression of the TGF-β gene in the lungs of the TCRγδ+/MDV+ was significantly higher than that in the MDV+ group and the TCRγδ-/MDV+ group at 10 dpi (*p* < 0.05), and lower than that in the control group at 21 dpi (*p* < 0.01) (Figure 12D). A significantly higher perforin gene expression was observed in the lungs of the TCRγδ-/MDV+ group compared to the control and the TCRγδ-/MDV- groups at 10 dpi (*p* < 0.05), but there was no significant difference in perforin gene expression between the TCRγδ+/MDV+ group and the control group (*p* ≥ 0.05) (Figure 12F).

## 4. Discussion

Chicken γδ T cells are suggested to be involved in immunity against MDV infection [5]. In the current study, we applied an autologous cell infusion method to investigate if TCRγδ-stimulated γδ T cells are involved in protective immunity against MD or in MDV pathogenesis. The ex vivo activation of circulating γδ T cells via TCRγδ stimulation and its effect on other PBMCs were analyzed. The results demonstrated that TCRγδ stimulation induces IFN-γ production from circulating γδ T cells including CD8^+^ γδ T cells, but this stimulation did not lead to TGF-β production or degranulation (Figure 1). It has been reported that IFN-γ produced by activated human γδ T cells initiates a T helper type 1 (Th1) response via the induction of Th1-promoting dendritic cells (DCs) [30,31,32]. We also demonstrated an association of the induction of a potent anti-MDV Th1 response with resistance to MD [33,34]. Our results showing that Th1 cytokines, IFN-γ and IL-2, were upregulated and one of the anti-inflammatory cytokines, TGF-β, was downregulated in ex vivo-stimulated PBMCs with anti-TCRγδ mAb and in the spleen after the infusion of ex vivo-stimulated PBMCs at the transcript level indicate that activated chicken γδ T cells may contribute to the initiation of the Th1 response, which is shown to be important for the control of MD-lymphoma (Figure 2). IFN-γ induces nitric oxide production and upregulates major histocompatibility complex (MHC) class II molecules in macrophages, and subsequently induces the activation of adaptive immune cells [35,36,37]. Recently, it was reported that IFN-γ inhibits MDV replication and interferes with MD progression [38]. Therefore, the upregulation of IFN-γ at the initial infection phase of the TCRγδ+/MDV+ group may contribute to preventing viral replication and subsequent MD progression. In our study, the results demonstrate that the protective role of adoptive γδ T cell transfer against MD may be attributed to IFN-γ production from immune system cells at the early phase of MDV infection (4 dpi) (Figure 10 and Figure 11). It is noteworthy to mention that IFN-γ production by γδ T cells at 4 dpi was not significantly higher in MDV-challenged chickens compared to the control chickens. In mammalian studies, it is known that the rapid production of IFN-γ by γδ T cells initiates activation of the cell-mediated immune response, which could contribute to the control of MD [39,40].

In the current study, we observed the upregulation of IL-17A gene expression in TCRγδ-stimulated PBMCs (Figure 2). As Walliser and Göbel reported, IL-17A is produced by αβ and γδ T cells upon PMA/ION stimulation in chickens, and γδ T cells may be one of the IL-17A-producing cells in response to TCR stimulation [41]. In mammals, γδ T cells immediately produce IL-17A and induce inflammation upon pathogen infections [42]. Thus, our results indicate that TCRγδ stimulation initiates both Th1 and Th17 responses. Considering that high expression of the IFN-γ gene was observed in the TCRγδ+/MDV+ group at 4 dpi, and IL-17 gene expression was downregulated at the similar level as the control group by 10 dpi, the immune response may have skewed toward the Th1 response as a result of MDV infection.

Cytotoxic activity by CD8^+^ T cells, NK cells, and γδ T cells is also suggested to be one of the critical factors in immune responses against MDV infection due to the cell-associated feature of MDV [5,10,13,43,44]. These cells are thought to induce apoptosis in MDV-infected cells via a granzyme–perforin pathway or a Fas–Fas ligand pathway [29,45]. In the present study, we observed an upregulation of granzyme A expression in the spleen, PBMCs, and lungs of the TCRγδ+/MDV+ group at 10 dpi (Figure 10, Figure 11 and Figure 12). Moreover, γδ T cells and CD8α^+^ γδ T cells in the TCRγδ+/MDV+ group demonstrated enhanced degranulation activity (Figure 8 and Figure 9). In mammals, herpes simplex virus (HSV) studies suggest that CD8^+^ T cells are retained in local infected sites and are able to be activated immediately at the early phase of reactivation [46]. These CD8^+^ T cells produce granzyme which inhibits the spread of HSV and viral reactivation from latency [47,48]. Therefore, the upregulation of cytotoxic activity at 10 dpi provided by the infusion of TCRγδ-activated PBMCs may constitute a rapid reaction against the early phase of MDV reactivation and may suppress MDV reactivation, which could prevent or delay MDV tumor formation. γδ T cells may also contribute to the suppression by cytotoxicity.

Although reduced virus replication in the local tissue and lower tumor incidence were observed in the TCRγδ+/MDV+ group, the infusion of TCRγδ-stimulated PBMCs did not affect virus shedding from feathers in the present study (Figure 3, Figure 4 and Figure 5). A previous MDV study suggested that even the chickens that are vaccinated, and are protected against tumor, shed the virus from feathers [28]. Our findings in the current study indicate that the infusion of activated γδ T cells is not sufficient to overcome the immunoregulatory environment in the skin of MDV-infected chickens [49,50,51].

CD25, which is also known as the IL-2 receptor α chain, is a T cell-activation marker as well as a marker of regulatory T cells in mammals [52]. Similar to mammals, CD25 is suggested as an activation marker of chicken T cells including γδ T cells [53,54]. In particular, a specific γδ T cell subset, CD8αα^+^ γδ T cells, upregulates CD25 expression upon *Salmonella* stimulation [53]. In a human study, IL-2-stimulated γδ T cells upregulated CD25 expression and enhanced CD25 expression when they received TCR stimulation [55]. However, in the present study, chicken γδ T cells cultured with IL-2 expressed CD25 more than γδ T cells which were cultured with IL-2 and anti-TCRγδ mAb (Figure 1). In addition to the ex vivo stimulation study, CD25 expression was decreased in γδ T cells in the TCRγδ+/MDV+ group at 10 dpi, though IFN-γ-producing γδ T cells were increased (Figure 7). Considering that chicken γδ T cells in the PBMCs and tissues are heterogeneous, CD25-expressing γδ T cells may be a different subset from IFN-γ-producing γδ T cells.

TGF-β gene expression reached a peak at 10 dpi in the spleen and lungs of the TCRγδ-activated PBMCs-infused groups, whereas it was decreased in the other groups from 4 dpi to 10 dpi regardless of MDV infection (Figure 10 and Figure 12). These results suggested that TGF-β expression was highly impacted by the infusion of TCRγδ-activated PBMCs. Since we observed the initiation of an inflammatory reaction upon the stimulation of TCRγδ in the current study, the upregulation of TGF-β in the spleen and lungs of the activated PBMCs-infused chickens at 10 dpi might be associated with the immune resolution of the acute inflammation [56]. In a mammalian study, it was reported that TGF-β in combination with IL-2 enhanced an increase in anti-apoptosis molecules and a decrease in pro-apoptosis molecules in γδ T cells [57]. As we observed TGF-β upregulation and no change or mild decrease in γδ T cell frequency in the spleen of the TCRγδ+/MDV+ group at 10 dpi in the present study, TGF-β may protect γδ T cells from apoptosis (Figure 6 and Figure 10).

Previous MDV studies revealed that challenging chickens with MDV upregulates IL-10 expression in the spleen, including in splenic CD4^+^ T cells, CD8^+^ T cells, and γδ T cells at the later phase of infection [14,28,36,58]. In the current study, both IL-10 and IFN-γ were upregulated in the spleen of MDV-challenged chickens, especially in the TCRγδ+/MDV+ group (Figure 10). The upregulation of both IL-10 and IFN-γ has been reported in human cancer, and it has been proven that IL-10 enhances IFN-γ and granzyme production in tumor-infiltrating CD8^+^ T cells [59]. Although a paradoxical role of IL-10 has been revealed in human cancer studies, the functions of IL-10 and the relationship between IL-10 and IFN-γ in MDV tumor formation remain to be elucidated [28,60].

IL-17A has both an anti-tumor and pro-tumor effect in tumor lesions [61]. IL-17A is produced by T helper type 17 (Th17), which is differentiated from naïve T cells in the presence of IL-6, IL-21, or IL-23 [62]. At the tumor site, IL-17 induces the production of angiogenic factors which accelerate microvessel generation. On the contrary, IL-17 induces the recruitment of DCs in a chemokine-dependent manner [62]. In mammals, IL-17-producing γδ T cells are involved in cancer immunity [63]. In the current study, the expression of IL-17A in the PBMCs of the TCRγδ+/MDV+ group was significantly lower than that of the TCRγδ+/MDV- group (Figure 11). A human cancer study revealed that the expression level of IL-17 in the PBMCs is correlated with cancer progression and IL-17 directly induces tumor proliferation [64]. Therefore, low IL-17A expression at 21 dpi provided by the infusion of activated PBMCs may be associated with the prevention of tumor formation by MDV.

In the current study, whole PBMCs were infused to chickens because chicken γδ T cells were not expanded after stimulation with anti-TCRγδ mAb, and, indeed, γδ T cell-expansion methods have not yet been established in chickens. The development of γδ T cell-expansion techniques will be required for further adoptive transfer studies in chickens. As an MDV study successfully investigated the B cell role in immunity against MDV using specific gene-knockout chickens, transgenic chickens that lack γδ T cells might be another method to examine the γδ T cell role in MDV-infected chickens [65].

In conclusion, TCRγδ stimulation induced significant IFN-γ production by chicken γδ T cells derived from the PBMCs, and decreased TGF-β gene expression in the TCRγδ-stimulated PBMCs. The infusion of TCRγδ-activated PBMCs decreased MDV replication in the lungs and prevented or delayed MDV tumor formation in MDV-challenged chickens. Activated γδ T cells may contribute to the initiation of immune responses against MDV at the early infection phase and control MD by inducing cytokines and cytotoxic activity during MDV infection.

## Figures and Tables

**Table 1:** List of antibodies for flow cytometry.

Target	Fluorochrome	Clone	Host Species	Source
CD3ε	Pacific Blue	CT-3	mouse	Southern Biotech
CD4	PE-Cyanine 7 or allophycocyanin	CT-4	mouse	Southern Biotech
CD8α	fluorescein-5-isothiocyanate	CT-8	mouse	Southern Biotech
TCRγδ	phycoerythrin	TCR-1	mouse	Southern Biotech
CD25	fluorescein-5-isothiocyanate	AbD13504	human	Bio-Rad Laboratories Inc. Hercules, CA, USA
IFN-γ	biotin	5C.123.08	mouse	Invitrogen
Streptavidin	allophycocyanin			Invitrogen
TGF-β	allophycocyanin	1D11	mouse	Bio-Techne, Inc. Minneapolis, MN, USA
CD107a	PE-Cyanine 7	LEP100 hybridoma cells	mouse	Developmental Studies Hybridoma Bank, Iowa City, IA, USA

**Table 2:** Primer sequences for real-time PCR.

Target Gene	Primer Sequence		Accession Number	Reference
β-actin	Forward	5′-CAACACAGTGCTGTCTGGTGGTA-3′	X00182	[23]
	Reverse	5′-ATCGTACTCCTGCTTGCTGATCC-3′		
meq	Forward	5′-GTCCCCCCTCGATCTTTCTC-3′	AY571783	[22]
	Reverse	5′-CGTCTGCTTCCTGCGTCTTC-3′		
gB	Forward	5′-GTCTGTTCAATTCGCCATGCTCC-3′	AY129966	[24]
	Reverse	5′-CCTTCCTAATGTTGCACTCGCTG-3′		
IFN-γ	Forward	5′-ACACTGACAAGTCAAAGCCGCACA-3′	X99774	[25]
	Reverse	5′-AGTCGTTCATCGGGAGCTTGGC-3′		
IL-2	Forward	5′-TGCAGTGTTACCTGGGAGAAGTGGT-3′	NM_204153.2	[26]
	Reverse	5′-ACTTCCGGTGTGATTTAGACCCGT-3′		
IL-12p40	Forward	5′-TTGCCGAAGAGCACCAGCCG-3′	AY262752.1	[25]
	Reverse	5′-CGGTGTGCTCCAGGTCTTGGG-3′		
IL-17A	Forward	5′-TATCAGCAAACGCTCACTGG-3′	NM_204460.2	[27]
	Reverse	5′-AGTTCACGCACCTGGAATG-3′		
IL-10	Forward	5′-AGCAGATCAAGGAGACGTTC-3′	AJ621614	[28]
	Reverse	5′-ATCAGCAGGTACTCCTCGAT-3′		
TGF-β	Forward	5′-CGGCCGACGATGAGTGGCTC-3′	NM_001318456.1	[25]
	Reverse	5′-CGGGGCCCATCTCACAGGGA-3′		
granzyme A	Forward	5′-TGGGTGTTAACAGCTGCTCATTGC-3′	NM_204457.2	[29]
	Reverse	5′-CACCTGAATCCCCTCGACATGAGT-3′		
perforin	Forward	5′-ATGGCGCAGGTGACAGTGA-3′	XM_046929135.1	[29]
	Reverse	5′-TGGCCTGCACCGGTAATTC-3′		

**Table 3:** Cycling parameters for real-time PCR.

Target Gene	Cycling Parameters					Efficiency
	Amplification				Melting Temp (°C)	
	Denaturation	Annealing	Extension	Number of Cycles		
β-actin	95 °C 10 s	58 °C 5 s	72 °C 10 s	40	97	2
meq	95 °C 10 s	64 °C 5 s	72 °C 8 s	45	97	1.8
gB	95 °C 10 s	64 °C 5 s	72 °C 8 s	45	97	1.93
IFN-γ	95 °C 10 s	60 °C 5 s	72 °C 10 s	50	97	1.98
IL-2	95 °C 10 s	58 °C 5 s	72 °C 10 s	50	97	2
IL-12p40	95 °C 10 s	64 °C 5 s	72 °C 10 s	45	95	2
IL-17A	95 °C 10 s	60 °C 5 s	72 °C 10 s	55	97	1.9
IL-10	95 °C 1 s	55 °C 5 s	72 °C 5 s	55	97	1.78
TGF-β	95 °C 10 s	60 °C 5 s	72 °C 10 s	40	97	2
granzyme A	95 °C 10 s	55 °C 5 s	72 °C 10 s	50	97	2.02
perforin	95 °C 10 s	64 °C 5 s	72 °C 10 s	50	97	1.91

## Data Availability

The data presented in this study are available from the corresponding author on reasonable request.

## Supplementary Materials

The following supporting information can be downloaded at: https://www.mdpi.com/article/10.3390/v15020285/s1, Figure S1: Gating strategies for flow cytometry.
