# Supplementary material for: Activated Chicken Gamma Delta T Cells Are Involved in Protective Immunity against Marek’s Disease
Source: Viruses. 2023 Jan 19;15(2):285. doi: 10.3390/v15020285 (PMC9962238; doi:10.3390/v15020285)
Supplement: Supplementary file 1 [file viruses-15-00285-s001.zip › viruses-2137907-supplementary.pdf]

Activated chicken gamma delta T cells are involved in protective immunity against Marek's disease

Ayumi Matsuyama-Kato, Bahram Shojadoost, Nitish Boodhoo, Sugandha Raj, Mohammadali Alizadeh, Fatemeh Fazel, Charlotte Fletcher, Jiayu Zheng, Bhavya Gupta, Mohamed Faizal Abdul-Careem, Brandon L. Plattner, Shahriar Behboudi, and Shayan Sharif

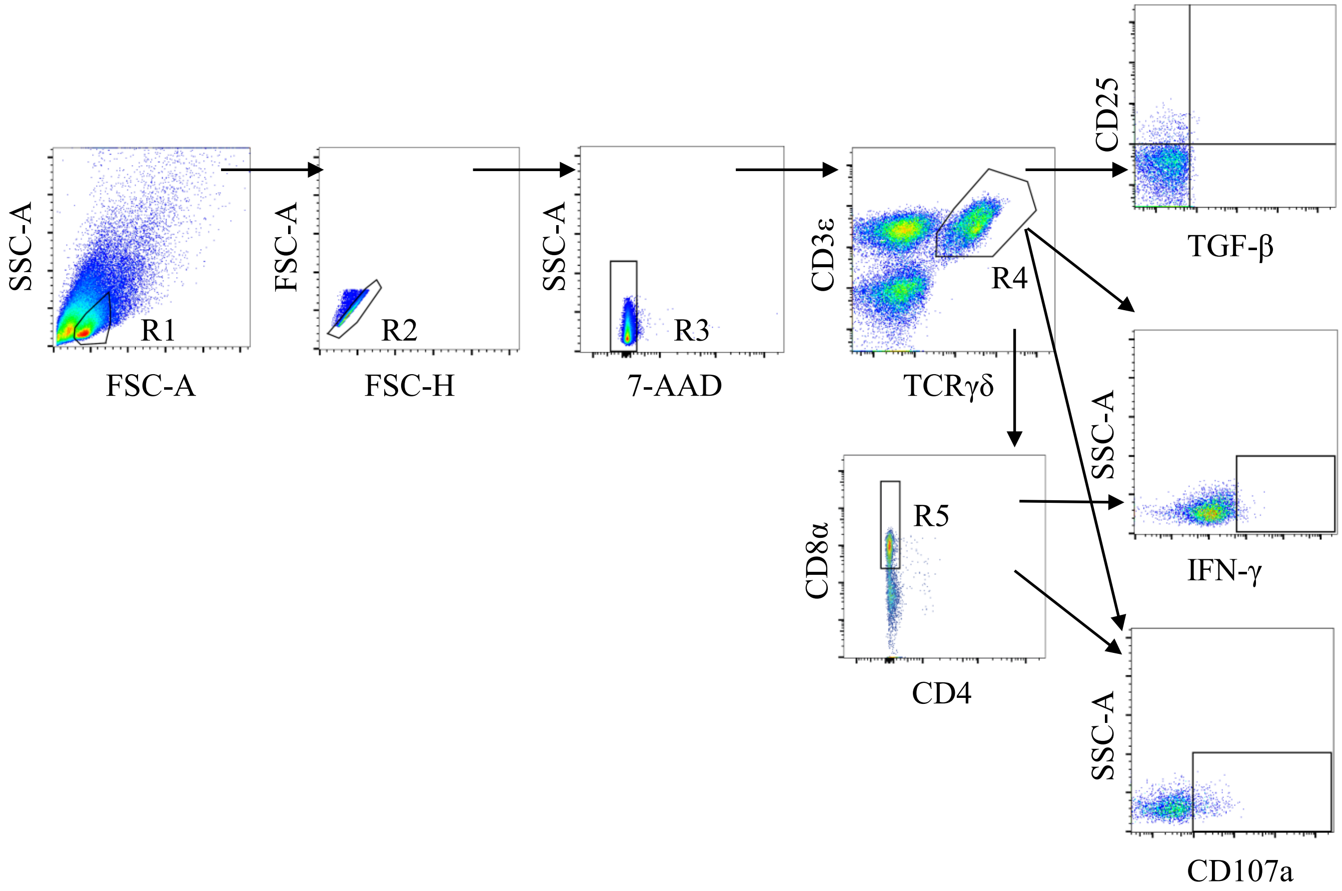

Supplementary Figure

Representative gating strategy is shown for defining IFN-γ^+ γδ T cells, TGF-β^+ γδ T cells, and CD107a$^+$ γδ T cells in live mononuclear cells by 7-AAD exclusion from the control, the TCRγδ-/MDV-, the TCRγδ+/MDV-, the MDV+, the TCRγδ-/MDV+, and the TCRγδ+/MDV+ groups (spleen at 4 dpi).
